# Polycyclic aromatic hydrocarbons in permafrost peatlands

**DOI:** 10.1038/s41598-021-98384-z

**Published:** 2021-09-23

**Authors:** Alexander Pastukhov, Sergey Loiko, Dmitry Kaverin

**Affiliations:** 1 Institute of Biology Komi Science Centre, Ural Branch, Russian Academy of Sciences, Syktyvkar, Russia; 2 National Research Tomsk State University, Tomsk, Russia

**Keywords:** Biogeochemistry, Environmental impact

## Abstract

The concentrations of 15 individual PAHs in 93 peat cores have been determined by using high-performance liquid chromatography methods. In the profile the qualitative and quantitative composition of PAHs was non-uniform estimated in a wide range: from 112 to 3673 ng/g with mean 1214 ± 794 ng/g. Among 15 identified individual PAHs, the main contribution to their total amount was made by heavy highly condensed PAHs in the Eastern European peat plateaus, in particular, 6-nuclear benzo[*ghi*]perylene (1021 ± 707 ng/g), whereas in West Siberian permafrost peatlands, light PAHs were dominating, mostly naphthalene and phenanthrene (211 ± 87 and 64 ± 25 ng/g, respectively). The grass-equisetum peat contained the maximum of heavy PAHs and the dwarf shrub-grass—the minimum. In grass-dwarf shrub, grass-moss and moss peat, the share of 2-nuclear PAHs was most significant: naphthalene and fluorene, as well as 6-nuclear benzo[*ghi*]perylene. The presence of benzo[*ghi*]perylene in the entire peat strata, including its permafrost layer, was a marker of the anaerobic conditions that persisted throughout the Holocene and they were necessary for the synthesis of this compound.

## Introduction

Peatlands, occupying only 3% of the land surface, contain about 15–30% of global soil organic carbon reserves^1^. The bogginess of the subarctic sectors of the European North-East and Western Siberia occupies a leading role in the climate regulation on the Earth.

The results of predictive modeling show that most of near-surface permafrost will thaw by the end of this century practically to the Barents and Kara seashores^2,3^, which will cause serious consequences for the functioning of ecosystems, especially at the southern permafrost limit, where the ground temperatures are already close to 0 °C^4,5^. Future permafrost thaw could lead to the release and inclusion of significant amounts of chemical elements and substances into geochemical flows^6,7^. This requires the understanding of what compounds are stored in permafrost.

Along with traditional methods (plant macrofossil and palynological analyses of peat), qualitatively new geochemical methods and approaches are now widely used, showing the response of vulnerable Arctic and subarctic ecosystems to the climate change^8^. Functional characteristics and indicators of the properties of peatlands were determined by using geochemical methods^9–13^. To identify the plant groups that compose peat layers, the analysis of different chemical parameters is widely applied: the composition of humic substances and low molecular weight organic compounds—aliphatic compounds, phenols, n-alkanes, alcohols, carbohydrates, low molecular weight organic acids^14–24^. These markers were used to analyze soil organic carbon of permafrost peatlands in the tundra and forest-tundra, located in zones of continuous and massive island permafrost distribution^25–28^.

However, these indicators cannot have a universal application due to the difference in species communities composing the peat, geochemical and landscape characteristics. It is necessary to search for reliable indicators of the decomposition degree of organic carbon, which can be used to track the effect of permafrost thaw on the organic matter preservation from mineralization, despite the differences in peat botanical composition. In our study, the composition of PAHs is considered as one of the indicators of peat plateaus/thermokarst complexes genesis^29^. 6-nuclear benz[*ghi*]perylene as the most immobile and stable in an anaerobic environment, but relatively rapidly decomposed under aerobic conditions, can be a sufficiently reliable bioindicator of the persistence of organic matter during the Holocene and in the future, even under permafrost thawing, because the anaerobic conditions will persist.

Polycyclic aromatic hydrocarbons (PAHs) are high molecular weight organic compounds, the main structural element of which is benzene (aromatic) rings connected with each other. 16 PAHs (including naphthalene, pyrene, phenanthrene, anthracene, and benzopyrene) have been identified as toxic pollutants by the United States Environmental Protection Agency (EPA)^30^; they have carcinogenic and mutagenic activity and pose a hazard to human health. At the same time, PAHs are widespread in the environment, since they are highly hydrophobic, inert to decomposition, and able to be adsorbed in soil and bottom marine sediments. PAHs are widely detected from Antarctica^31,32^ to dry^33,34^ and humid^35^ tropical areas in various types of environmental compartments including marine organisms^36^.

PAHs are formed both from natural sources: fires, oil seepage, biogenic chemical reactions, and anthropogenic emissions as a result of fossil fuel combustion and atmospheric transport with precipitation of the PAHs^37^. If mineral soils, including their organogenic horizons, contain small amounts of natural PAHs of various genesis, the peatlands can actively accumulate PAHs. Peat is a product of biogenic accumulation, and a peat deposit is a chronicle pattern of changing flows of organic compounds, including PAHs of natural and anthropogenic origin^38^. If organic matter in permafrost peat plateaus is conserved, it becomes vulnerable to mineralization during permafrost thaws. PAHs are characterized by high chemical stability, which, in combination with acidic and anaerobic cathotelm environment, does not result in PAH degradation. Thus, it is necessary to study the quantitative and qualitative PAH composition, especially in permafrost peat plateaus, conserving huge stocks of potentially labile organic carbon.

There has been a high scientific interest in PAH study over the past decades^32,33,39–45^, however, studies of the features of PAH accumulation in peatlands are extremely underrepresented^46–48^, and in permafrost peatlands, they are singles^29,49,50^.

The purpose of this study was to assess the profile distribution of PAHs in permafrost peatlands in the East European and West Siberian permafrost zones.

The objectives of the work are: (1) to compare of the PAHs profile distribution patterns in permafrost peatlands of different botanical composition; (2) to identify of peat deposits having maximum PAH accumulation; (3) to determine of trends in the accumulation of various PAH groups; (4) to compare of PAH accumulation in permafrost peatlands of the East European and West Siberian plains.

## Materials and methods

### Site description

**Figure 1:**
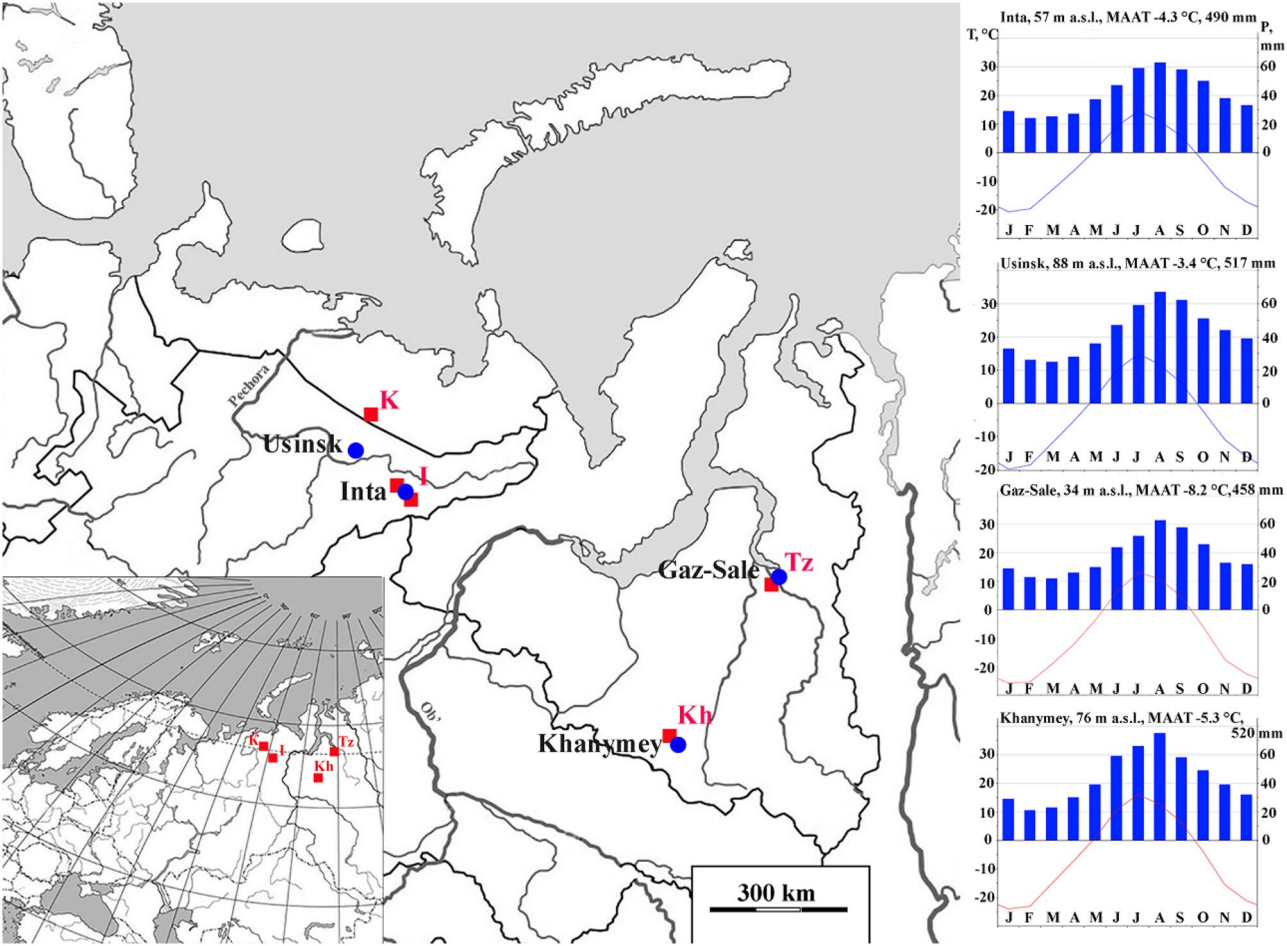
Map showing the location of the sampling study sites (indicated by red squares). The closest climate stations indicated by blue circles. Monthly air temperatures and precipitation based on the climate database https://ru.climate-data.org/.

The study area is located in permafrost peat plateau/thermokarst complexes (polygonal and uplifted mounds and fens), from the tundra to the northern taiga in the cryolythozone of the European Northeast and Western Siberia (Fig. 1). Mean annual air temperatures vary from − 3.4 to − 8.2 °C, mean annual precipitation is 450–520 mm^51^. Most of the study area is generally flat plain, with elevations from 40 to 160 m a.s.l., underlain by Upper Pleistocene glacial and lacustrine deposits^51^. A rather flat topography and shallow occurrence of permafrost table favors a significant waterlogging of the area. Shallow thermokarst streams and lakes are widespread, eolian and abrasion processes are developed, which mostly occur on steep slopes, river and lake banks.

To study peat plateau/thermokarst complexes, we selected representative key sites located in similar permafrost peatlands. Two sites (TZ and K) are located in the southern tundra, the other two sites (Kh and I) are in the northern and extreme northern taiga, respectively. Polygonal peatland (TZ—"Tazovsky": TZ 1, TZ 2 and TZ 3—67°20′N, 78°56′E) was investigated in the subzone of the southern tundra in Western Siberia with polygonal-roller soil complexes. In the northern taiga of Western Siberia (Kh—"Khanymei": Kh 2 and Kh 3—63°42′N, 75°54′E), in the southern tundra (K—“Kolva”: K 1 and K 2—63°42′N, 75°54′E) and in the extreme northern taiga (I—"Inta": I 1—65°54′N, 60°26′E; I 2—65°25′N, 60°49′E; I 3—66°04′N, 60°05′E; I 4—66°00′N, 60°05′E; I 11—66°05′N, 59°58′E) in the European Northeast, areas of peat plateaus with shallow permafrost tables are interspersed with permafrost free fens and thermokarst lakes open or in-filled with vegetation. According to the WRB^52^, the soils of polygons and peat plateaus are classified as Ombric Sapric Cryic Histosols (Hyperorganic), which characteristic diagnostic features are the presence of well-decomposed organogenic material (peat) down to more than 2 m deep, predominantly atmospheric nutrition and the underlying permafrost within 1 m. The soils of the cracks are classified as Hemic Muusic Histosols, where the moderately decomposed peat accumulates on the polygonal-veined ice filling the cracks; soils of seasonally freezing fens belong to Hemic Histosols. Permafrost is absent or located deeper than 10 m in fens (the maximum depth of borehole drilling). The soils of the peat circles (bare surfaces on permafrost peatlands) are identified as Ombric Sapric Cryic Histosols (Hyperorganic Turbic), because the upper horizons have signs of peat cryoturbation. These bare peat circles are round patches with a diameter of 4–25 m and areas from 10 to 500 m^2^, covering up to 1–10% of the total peat plateau.

The upheaved peat mounds (up to 2–3 m high) are drier than the peat circles and are sparsely vegetated. Unlike palsas, the peat mounds have no isolated frozen cores, being the extensions of the permafrost peat plateau. The mounds might often be oval in shape, elongated in the northwest–southeast direction by dozens of meters. The polygonal peatland is almost flat from the surface, the excess of polygons over depressions (cracks) is up to 0.5 m. The main part of mounds and polygons is vegetated with polydominant shrub-lichen communities, e.g., *Betula nana* L., *Ledum palustre* L., *Vaccinium vitis-idaea* L., *V. uliginosum* L., *Empetrum hermaphroditum* L., *Rubus schamaemorus* L., Bryidae mosses (*Dicranum*, *Polytricum*) and lichens (mainly representatives of the genus *Cladonia*). Shrub-Eriophorum-Sphagnum phytocenoses are widespread in fens and interpolygonal depressions. The herb-dwarf shrub vegetation is dominated by *Betula nana* and *Eriophorum media*, as well as *Rubus chamaemorus*, *Oxycoccus microcarpus*. The ground layer is covered by *Sphagnum fuscum*, *S. compactum*, etc.

Thus, the study area covers a vast part of the cryolithozone: from the Tazovsky site with a predominantly continuous permafrost 250–450 m thick and a shallow active layer (< 0.5 m), where open taliks are located only under the channels of large rivers and deep lakes: the upper horizons of the permafrost have low mean annual ground temperatures (MAGT) varying from − 3 to − 7 °C and a significant distribution of syngenetic sediments with a volumetric ice content up to 40–60%, often in the form of thick ice-ground veins; to the Inta site, located at the extreme southern limit of the cryolithozone with sparsely island permafrost 0–25 m thick and MAGTs between 0 and − 0.5 °C^53,54^.

### Soil sampling and laboratory analyses

The peat soils were described and sampled in the center of peat mounds and polygons, as well as in the adjacent fens and inter-polygonal cracks. Soil samples were collected with a vertical resolution of 5–10 cm. In most of the sites the reference sampling depth matched with full peat strata. In Seida, Inta and Kolva sites deep boreholes (10 m) were drilled using a machine tool UKB 12/25-02 "Pombur".

The quantitative chemical analytical analyses of the soils were performed in the Center for Collective Use “Chromatography” of the Institute of Biology (Komi Science Center, Ural Branch of the Russian Academy of Sciences). The concentrations of PAHs in the soil samples were determined according to the US EPA method 8310^55^ and according to the method accepted by the Russian service for ecological monitoring^56^. Despite low detection limits and popularity of GC/MS–MS method, qualitative and quantitative PAH content in peat samples was determined by the method of reversed-phase high-performance liquid chromatography in a gradient mode and spectrofluorimetric detection on a liquid chromatograph "Lumakhrom". 15 different PAHs were totally identified. PAHs were completely extracted from peat using a Dionex ASE-350 Accelerated Solvent Extraction system (Thermo Fisher Scientific, USA). The ASE approach meets all international standards for extraction: U.S. EPA SW-846 3545^57^ an approach for extraction under the pressure, USEPA Contract Laboratory Program: Statement of Work for Organics Analysis: Multi-Media, MultiConcentration, OLM04.2.33. A peat sample weighting 1 g was put into a 100 cm^3^ flask and extracted by a mixture of acetone:hexane (1:1) three times under ultrasonic extraction in portions of 10 cm^3^.UV processing was carried out during 3 min for each extraction stage. The obtained extracts were concentrated by the Kuderna–Danish (K–D) apparatus to volume of 1–2 cm^3^,then 3–5 cm^3^ of n-hexane was added and the extracts were dried to 1–2 cm^3^ volume (but not to the absolute dryness). Extracts separating was carried out by using column chromatography in order to remove impurities interfering to define the PAHs. To separate the fractions, aluminum oxide and the second activity level silica gel according to Brokman were used (silica gel 60 for the column chromatography, Fluka, particle size 0.06–0.20 mm). The obtained effluents were concentrated by the K–D concentrator to volume of 1–2 cm^3^, then 3 cm^3^ of acetonitrile was added and the extracts were dried to 1–2 cm^3^. The sample concentrates were analyzed by the reversed phase HPLC in a gradient mode and spectrofluorimetrical detection (Fluorat-02-Panorama, Lumex, Russia). The solutions with a known content of PAHs were prepared by dilution of the standard solution of 15 PAHs (Fluka) with 10 mcg (cm^3^)^−1^ of each component. The accuracy of measurements and the PAH definition for peat soils were controlled using Standard Reference Material 1944 “New York/New Jersey Waterway Sediment” (National Institute of Standards and Technology, USA). The limits of the relative error, depending on the measurement range (with P = 0.95, ± δ, %), are for: naphthalene—16–50, acenaphthene—20–40, fluorene—18–40, phenanthrene—20–50, anthracene—18–50, fluoranthene, pyrene—18–46, benzo[*a*]anthracene—20–42, chrysene—22–52, benz[*b*]fluoranthene—22–42, benz[*k*]fluoranthene—18–48, benz[*a*]pyrene—18–50, dibenz[*a,h*]anthracene—20–48, benzo[*ghi*]perylene, indeno[*1,2,3-cd*]pyrene—22–44. Standard deviation of unit value from the measured value calculated for the Standard Reference Material was 3–25% (Statistica 10, Statsoft). The permissible value limit of mean square error for the output signal (n = 5) was 4%. Recoveries ranged from 80 to 95% for the reported PAHs in peat samples.

The analytical data were processed using the Microsoft® Excel 2010 software package and a statistical package R for data processing in ecology^58^. To study the relationship between PAHs and indicators of the peat physicochemical properties, a matrix of similarities was performed based on the Pearson correlation coefficients.

## Results

### Soil properties

Previous studies revealed the heterogeneity of the composition and properties of peat organic matter both in the active layer and permafrost^59,60^, indicating different paleogeographic conditions of their formation^23,61^.

**Figure 2:**
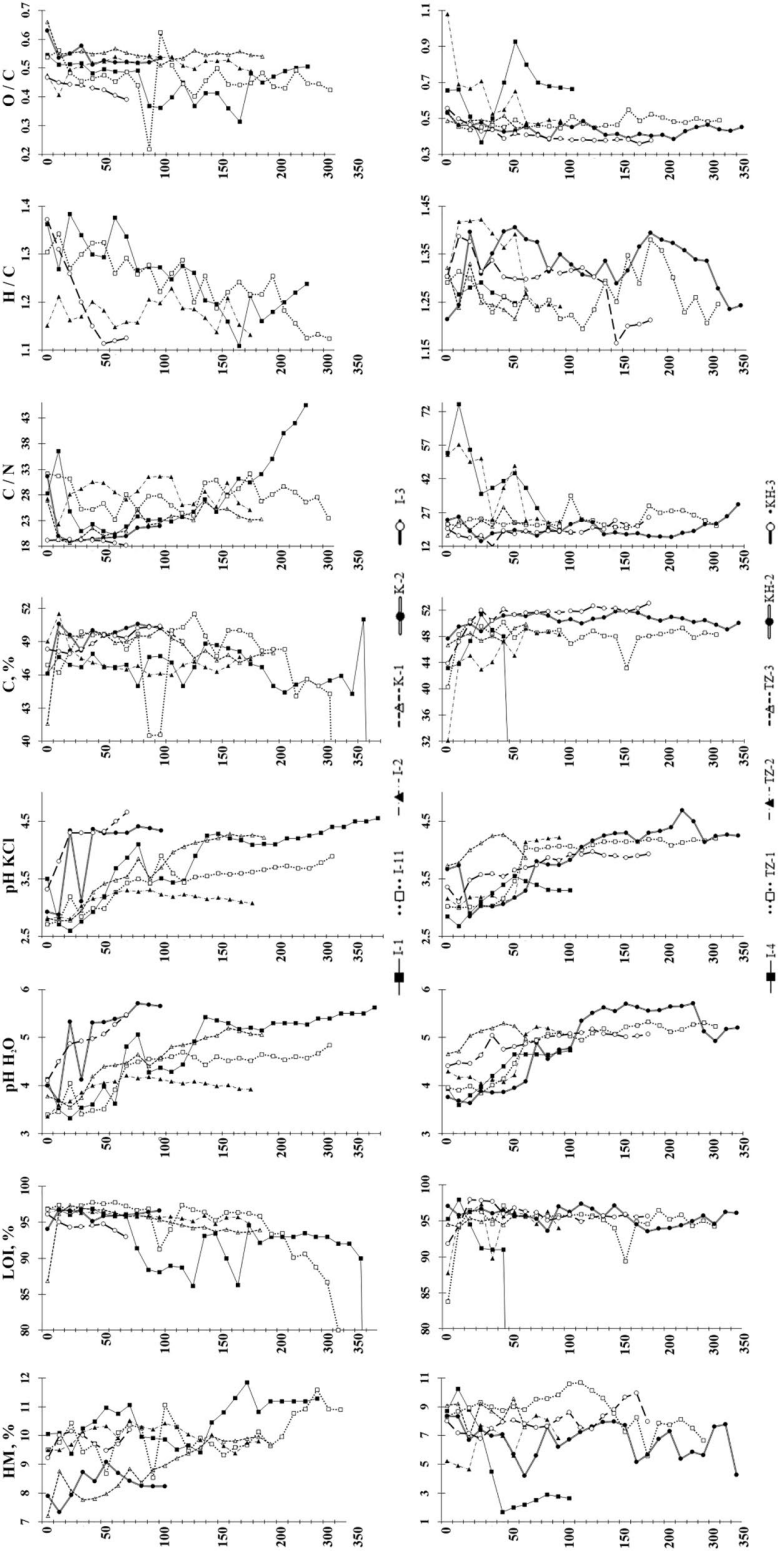
Physicochemical properties of the studied peat plateaus. HM, %—humidity moisture; LOI, %—loss on ignition.

At the peat plateaus of the European Northeast pH H_2_O and pH KCl are lower, 4.4 ± 0.51 and 3.5 ± 0.47, whereas pH values at West Siberian permafrost peatlands are 4.7 ± 0.47 and 3.7 ± 0.44, respectively. The pH values increase down the profile. The content of total organic carbon is 48.5 ± 1.78%, whereas its minimum values are explained by the presence of weakly decomposed wood residues in the peat. Nitrogen concentrations are estimated in a wider range from 0.79 to 4.9% with mean 2.49 ± 0.50% (Fig. 2).

A difference in the gross N mineralization of peat deposits results from the diversity in the peat characteristics, particularly in the C/N ratio^62^. There are three probable reasons for the low C/N ratio of the peat: the advanced stage of decomposition, as N is enriched relatively to carbon when peat is decomposed^63^; the possible minerotrophic origin of the peat^64^; or occurrence of unvegetated peat surfaces (peat circles) with very high N_2_O emissions (up to 1.40 ± 0.15 g N_2_O m^−2^ year^−1^). Obviously the similar situation has been during the Holocene^65^. When frost heave affects the peat strata it becomes vulnerable to winter abrasion caused by wind-drifted ice crystals, which can then remove the ombrotrophic peat, exposing the fen-origin peat to the surface. The C/N ratio is rather differentiated in the peat plateaus, varying from 12 to 75 with mean 25 ± 5.7. The highest C/N ratios are usually detected in the unfrozen near-surface horizons (0–20 cm), except for peat circles (profile TZ 3) and/or eroded upper peat layers (I 11). In the lower part of the active layer and the transition layer (45–75 cm), the C/N ratios are minimal 12–31. The transition layer is a layer between active layer and permafrost, which can presumably thaw in some warm years. In permafrost the C/N ratio gradually increases down the profile, except for horizons with multiple weakly decomposed remains of shrub-wood vegetation.

The H/C atomic ratio with mean 1.27 ± 0.06 indicates the predominance of aliphatic chains in molecules of humic acids in the presence of 40–50% aromatic structures. The oxidation state (O/C) is 0.49 ± 0.06, marking the predominance of oxidation processes only in the active layer.

The differences in physicochemical properties indicate changes of paleogeographic environments during the development of studied permafrost peatlands.

### Concentrations and distribution of PAHs in peatlands

**Figure 3:**
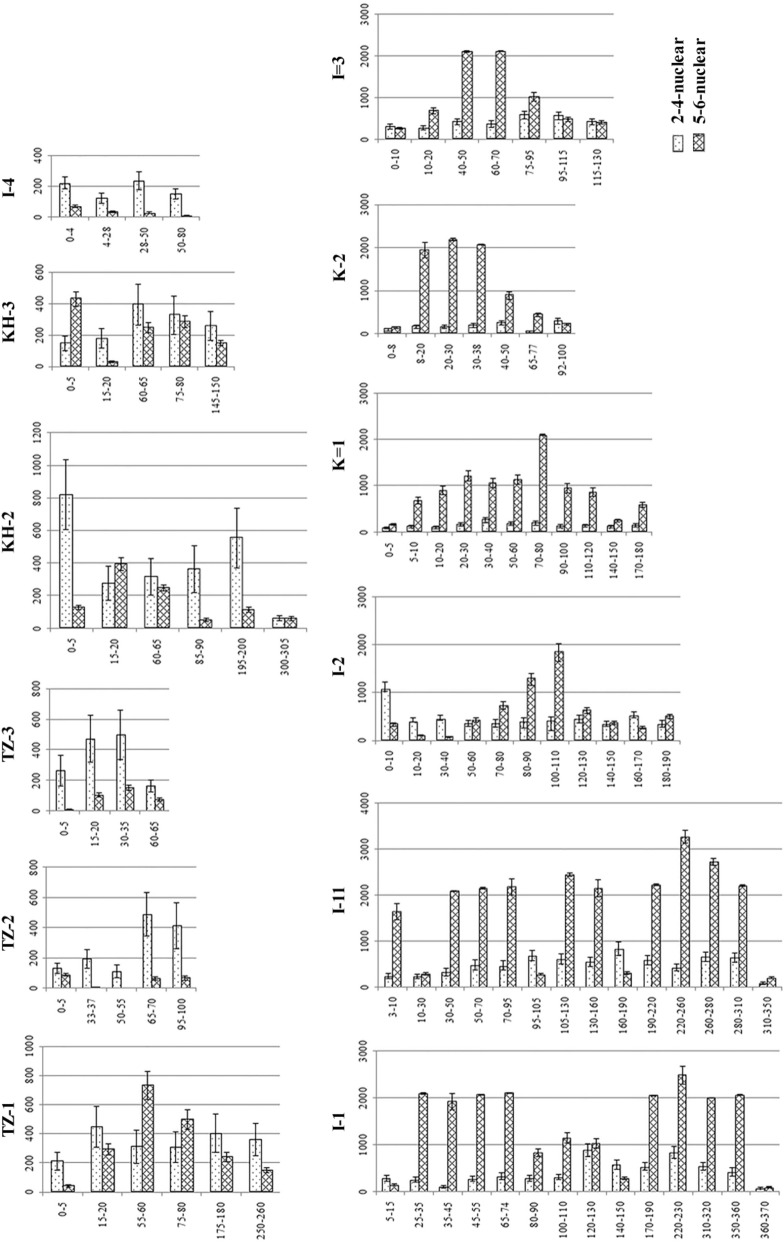
Distribution of low molecular weight (light) and high molecular weight (heavy) PAHs in peat plateaus.

Both the content of individual PAHs and their total concentration vary widely, from 112 to 3673 ng/g, with a mean value of 1214 ± 794 ng/g. Nevertheless, the contribution of individual PAHs to the total amount is not the same and depends on the concentration of heavy 5–6-nuclear polyarenes and, mainly, 6-nuclear benzo[*ghi*]perylene, the content of which in some peat layers exceeds the upper limit of determination—2000 ng/g (Fig. 3). When 6-core indeno[*1,2,3-cd*]pyrene is not detected anywhere, then all analyzed peat plateaus with a peat thickness ˃ 1 m located in the European Northeast have high concentrations of benzo[*ghi*]perylene, while in the West Siberia benzo[*ghi*]perylene is determined only in the upper layers of *Sphagnum* and *Scheuchzeria* peat in interpolygonal cracks and fens, and is low in content, only from 82 ± 20 to 377 ± 91 ng/g.

There are noticeably few 5-nuclear PAHs. Dibenz[*a,h*]anthracene is marked through the highest content with a mean value of 86 ± 89 ng/g in all the profiles, but its concentrations differ significantly: from being absent in relatively shallow peat deposits in the extremely northern taiga of the European Northeast (profiles I 3 and I 4) to its share in some horizons in the polygonal peatland of Western Siberia exceeding the sum of all other polyarenes, reaching 727 ± 220 ng/g. The portions of the remaining 5-nuclear PAHs (benzo[*b*]fluoranthene, benz[*k*]fluoranthene, and benzo[*a*]pyrene) are very low, with the exception of some extreme northern taiga peat plateaus in the European Northeast. At profile I 11, the content of benzo[*b*]fluoranthene increases up to 280 ± 70 ng/g, and in profile I 3, the amount of benzo[*k*]fluoranthene is 94 ± 23 ng/g.

In contrast to high-molecular-weight heavy PAHs, the concentrations of low-molecular-weight light PAHs are quite homogeneous at almost all the studied sites. Among 2-nuclear PAHs, naphthalene predominates (129 ± 68 ng/g), whereas in the European Northeast peatlands as compared to West Siberian ones fluorene concentrations are noticeably higher and equal 139 ± 70 and 10 ± 3 ng/g, respectively. The largest input to the sum of 3-nuclear PAHs is composed by phenanthrene, varying between 13 and 160 ng/g with a mean value 61 ± 31 ng/g, the anthracene content is mostly negligible. The concentrations of 4-nuclear PAHs are widely varying at the studied sites. The benzo[*a*]anthracene contribution is almost zero, whereas fluoranthene, pyrene and chrysene can also be absent or very low, with the exception of few horizons. Pyrene and chrysene contents reach several tens and even hundreds of ng/g—up to 260 ± 90 and 400 ± 110 ng/g, respectively.

Our results show that the vertical distribution patterns of light 2–4-nuclear and heavy 5–6-nuclear PAHs are differentiated. Thus, in all the analyzed peatlands of the European Northeast, with the exception of the shallow peatlands (peat thickness is 80 cm, e.g. profiles I 3 and I 4), the concentrations of heavy PAHs are 4.4 ± 2.8 times higher than the content of light polyarenes. At the same time, no significant regularity is observed in the mass fraction increase of high molecular weight PAHs down the profile. E.g., the maximum content of heavy PAHs is recorded at the boundary between the active layer and permafrost in profiles K 1, K 2, and I 3, but at profiles I 1, I 11, and I 2, the maximum of 5–6-nuclear PAHs is found mainly in deeper permafrost layers. On the contrary, in Western Siberia the concentrations of 2–4-nuclear light PAHs are 4.0 ± 3.4 times higher than those of highly condensed 5–6-nuclear polyarenes.

For testing the significant differences of PAH concentrations, the ANOVA test has been applied. By the results of the ANOVA test, 2–4, 5–6 nuclear PAHs and their total concentrations are differentiating both for individual peatland profiles and for key sites in general (Table 1). That is, these parameters are geographic.

**Table 1:** ANOVA results of the significant differences of PAH concentrations in the studied peat plateaus.

Effect^1^	2–4 PAHs					5–6 PAHs					Summary				
	SS	DF	MS	F	p	SS	DF	MS	F	p	SS	DF	MS	F	p
Profile	**1,281,972**	**11**	**116,543**	**3.777**	**0.000**	**31,667,885**	**11**	**2,878,899**	**6.300**	**0.000**	**37,625,931**	**11**	**3,420,539**	**6.678**	**0.000**
Error	2,529,723	82	30,850.3			37,471,223	82	456,966.1			41,996,082	82	512,147.3		
Key site	**933,532**	**4**	**233,383**	**7.216**	**0.000**	**21,429,779**	**4**	**5,357,445**	**9.994**	**0.000**	**23,879,619**	**4**	**5,969,905**	**9.532**	**0.000**
Error	2,878,163	89	32,338.9			47,709,329	89	536,059.9			55,742,395	89	626,319.0		

^1^Significant effects (p < 0.05) are in bold.

To verify the existence of a statistically significant difference between PAH concentrations, Post hoc analysis has been then performed using the Mann–Whitney U-test for key sites. Table 2 shows the results of the test. In terms of the content of 2–4 nuclear PAH, the peat cores of the Kolva key site are strongly distinguished. The site Kolva (profiles K 1 and K 2) differs from all other key areas, indicating the unique features of the peat massifs of this territory associated with the specificity of soil formation processes, which were described in detail by us earlier^60^. Significant differences in 5–6 nuclear PAH concentrations are shown in the West Siberian and East European key sites. Moreover, two key sites in Western Siberia, Tazovsky (profiles TZ 1, TZ 2, TZ 3) and Khanymei (Kh 2, Kh 3), despite the 420 km spread between them, does not show significant differences in concentrations, associated with the spatial homogeneity of both the underlying parent material and the relief, and, consequently, the peat of the West Siberian Plain. Key sites of the East European Plain do not show significant differences among themselves. Even Kolva is not reliably distinguishable from Inta (I 1, I 2, I 3, I 4, I 11). By the total amount of PAHs, in addition to the indicated differences, a significant difference is added between Kolva and Inta sites.

**Table 2:** Mann–Whitney U-test for (a) 2–4 nuclear PAH, (b) 5–6 nuclear PAH, (c) total amount of PAHs.

Key site	Tz	Kh	Inta	Kolva
**(a)**				
Tz		p = 0.917; U = 80	p = 0.158; U = 219	p = **0.000**; U = **40**
Kh	p = 0.917; U = 80		p = 0.271; U = 167	p = **0.003**; U = **33**
Inta	p = 0.158; U = 219	p = 0.271; U = 167		p = **0.000**; U = **89**
Kolva	p = **0.000**; U = **40**	p = **0.003**; U = **33**	p = **0.000**; U = **89**	
**(b)**				
Tz		p = 0.350; U = 64	p = **0.000**; U = **81**	p = **0.000**; U = **21**
Kh	p = 0.350; U = 64		p = **0.001**; U = **74**	p = **0.000**; U = **19**
Inta	p = **0.000**; U = **81**	p = **0.001**; U = **74**		p = 0.315; U = 292
Kolva	p = **0.000**; U = **21**	p = **0.000**; U = **19**	p = 0.315; U = 292	
**(c)**				
Tz		p = 0.604; U = 72	p = **0.000**; U = **99**	p = **0.003**; U = **53**
Kh	p = 0.604; U = 72		p = **0.001**; U = **80**	p = **0.013**; U = **43**
Inta	p = **0.000**; U = **99**	p = **0.001**; U = **80**		p = **0.047**; U = **235**
Kolva	p = **0.003**; U = **53**	p = **0.013**; U = **43**	p = **0.047**; U = **235**	

p < 0.05 are in bold.

**Figure 4:**
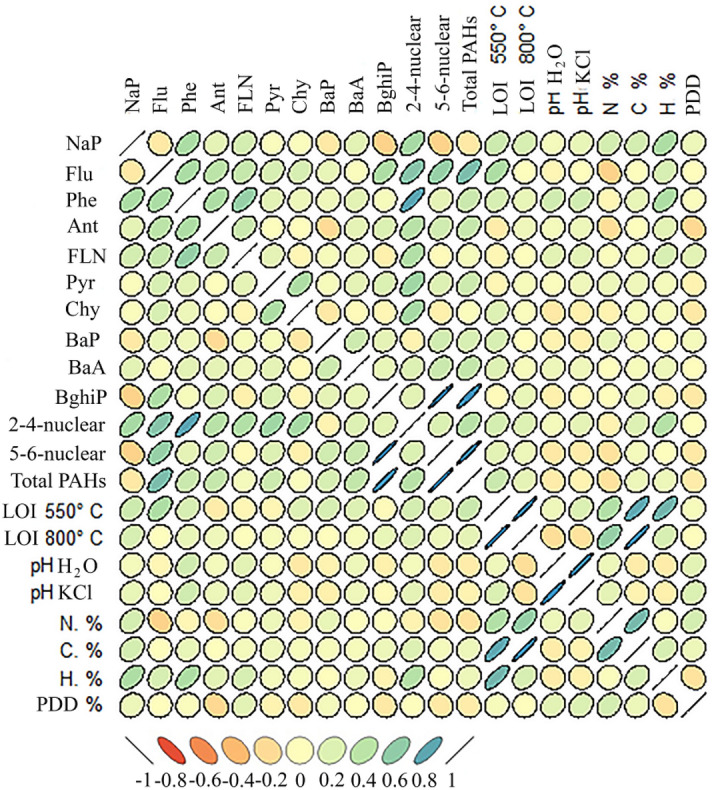
Coefficients of Pearson correlation between the PAH content and main indicators of the physicochemical properties of peat. PDD—peat decomposition degree, %.

A correlation analysis was performed and a matrix of Pearson correlation coefficients was constructed to study the interdependence between individual PAHs, the sums of 2–4-nuclear and 5–6-nuclear hydrocarbons and their total concentration with different physicochemical peat properties (Fig. 4). Because of the 15 identified PAHs, the concentrations of benzo[*a*]anthracene, benzo[*b*]fluoranthene, benzo[*k*]fluoranthene, and indeno[*1,2,3-cd*]pyrene are zero or extremely low, the calculations and discussions of results are based on 10 remaining PAHs.

The sum of 5–6-nuclear polyarenes and the total amount of PAHs depends on the concentration of benzo[*ghi*]perylene by 98 and 95%, respectively (Fig. 4). The amount of light PAHs mainly correlates with the content of phenanthrene and fluorene (R = 0.8 and 0.66, P < 0.01, respectively). Positive average correlations were found between the amount of phenanthrene with naphthalene—R = 0.51, P < 0.05, fluorene—R = 0.52, P < 0.05, and fluoranthene—R = 0.59, P < 0.05, as well as between chrysene and pyrene—R = 0.5, P < 0.05.

There are significant correlations between the content of light PAHs and the amount of C and N (R = 0.21 and 0.49, respectively, P < 0.05, total number of samples n = 91). Very weak and weak positive relationships are recorded between the content of naphthalene and the content of C, N, H, loss on ignition at 800 °C, which are 0.27; 0.28, 0.48 and 0.25, respectively. Weak correlations of the fluorene concentration are found with LOI at 550 °C (R = 0.49, P < 0.05), N (R = -0.25, P < 0.1) and H (R = 0.25, P < 0.1), as well as the phenanthrene content correlated positively with pH H_2_O and pH KCL (R = 0.28 and 0.29, respectively, P < 0.1).

**Figure 5:**
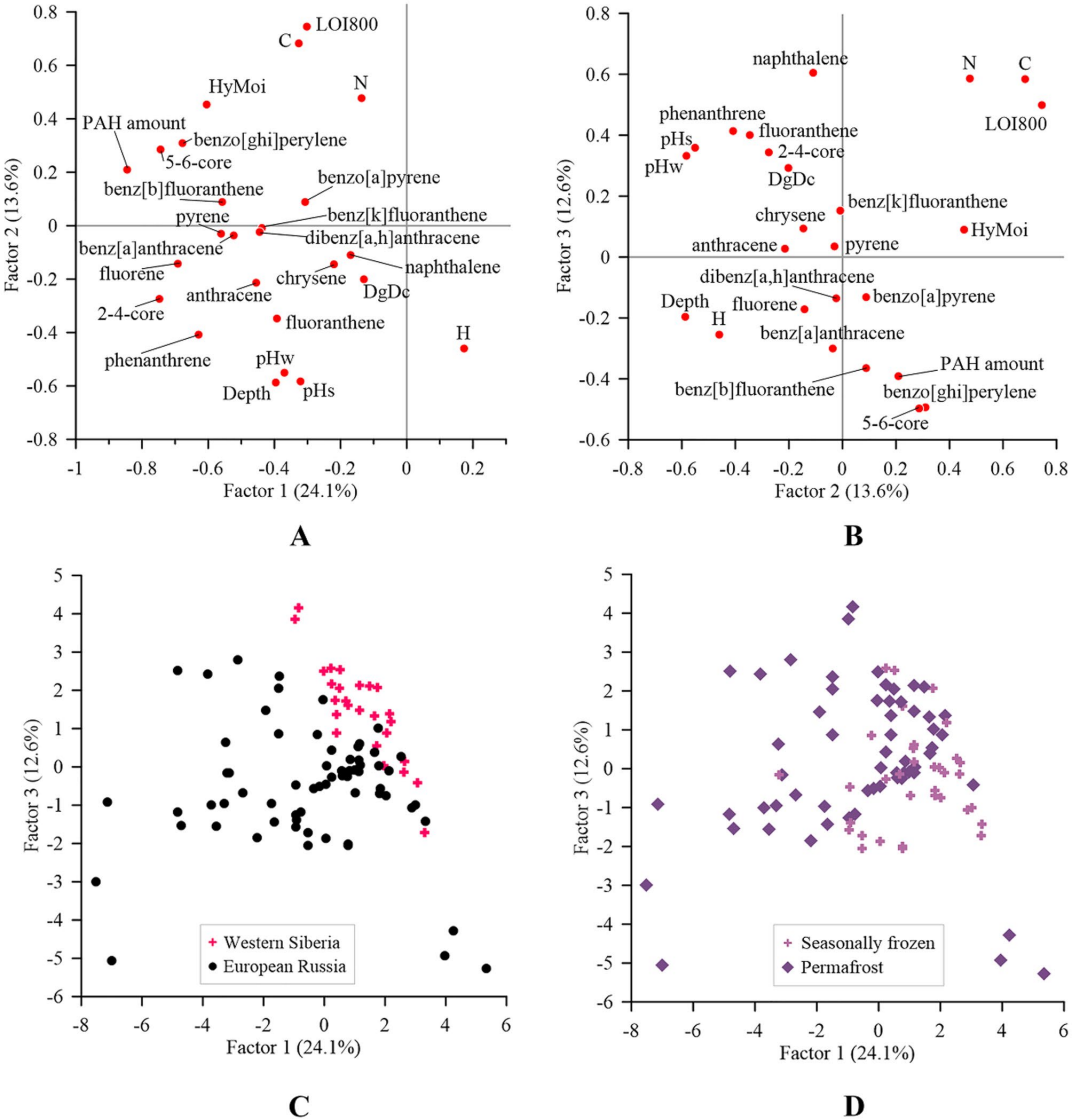
Results of PCA for peat layers. (**A**, **B**) Plots of F1 × F2 and F2 × F3 factors, (**C**) plot of F1 and F3 ordination of samples by region of selection, (**D**) plot of F1 and F3 ordination of samples for freezing regime.

To reduce the dimensionality of the data while retaining most of the variation in the data set, we use Principal component analysis (PCA) (Fig. 5).

By the results of PCA, PAH amount, 2–4-nuclear PAHs, 5–6-nuclear PAHs, fluorene, benzo[*ghi*]perylene, phenanthrene and humidity moisture (HyMoi) are negatively correlated with the first factor. These indicators have a correlation less than –0.6 with the first factor. They are the total PAH values. According to the same first factor, the samples from Western Siberia and the East European Plain are also divided, as the content of PAHs in the West Siberian samples is lower than in the soils of the East European Plain.

## Discussion

### The factors of the non-uniform distribution and very wide range of the PAH concentrations in the studied peatlands

By the previous studies, high molecular weight PAHs have a higher chemical reactivity compared to light low molecular weight polyarenes, and the most active transformation of PAHs occurs in acidic soils^66^. The range of pH KCl values in the studied polygonal peatlands and peat plateaus is within 2.68–4.66, however, there is no significant correlation with the concentrations of individual PAHs or sum of light or heavy PAHs.

The results reported here, based on peat plateaus in European Northeast (sites K 1, К 2 and I 3), confirm our previous findings that the PAH concentration is much lower in the active layer of peat plateaus, than in those in permafrost, which indicates the onset of aerobic conditions and ongoing biodegradation^29^. A significant increase in PAH content is due to highly condensed 5–6-nuclear polyarenes, and especially anomalously high concentrations of 6-nuclear benzo[*ghi*]perylene, at the transitional layer, the border between the lower active layer (35–60 cm) and the upper permafrost (60–80 cm), in the peat plateaus of the European forest-tundra^67^. The quantitative and qualitative analysis of PAHs at the boundary between the active layer and the upper permafrost can be used as a response indicator of permafrost on climate change at high latitudes, since the permafrost degradation leads to the transformation of preserved plant residues, humic substances, nonspecific organic compounds, and accumulation of heavy PAH structures^67^. However, in the European key areas I 1 and I 11, such a pattern is not expressed, and in the upper aerobic horizons of the active layer, the PAH concentrations are quite high, and in the upper horizons of the West Siberian sites, the content of light polyarenes can be maximum compared to the lower ones. Obviously, this fact testifies to the long-term preservation in the anaerobic conditions of the cathotelm and the conservation of the organic matter in the peat deposits and the relative "youth" of the peat. We earlier noted that light PAHs predominate in the recently formed (several hundred years) peat deposits, whereas heavy PAHs, especially 6-nuclear benzo[*ghi*]perylene, accumulate in large quantities only in ancient (several thousand years) peat^29^.

**Figure 6:**
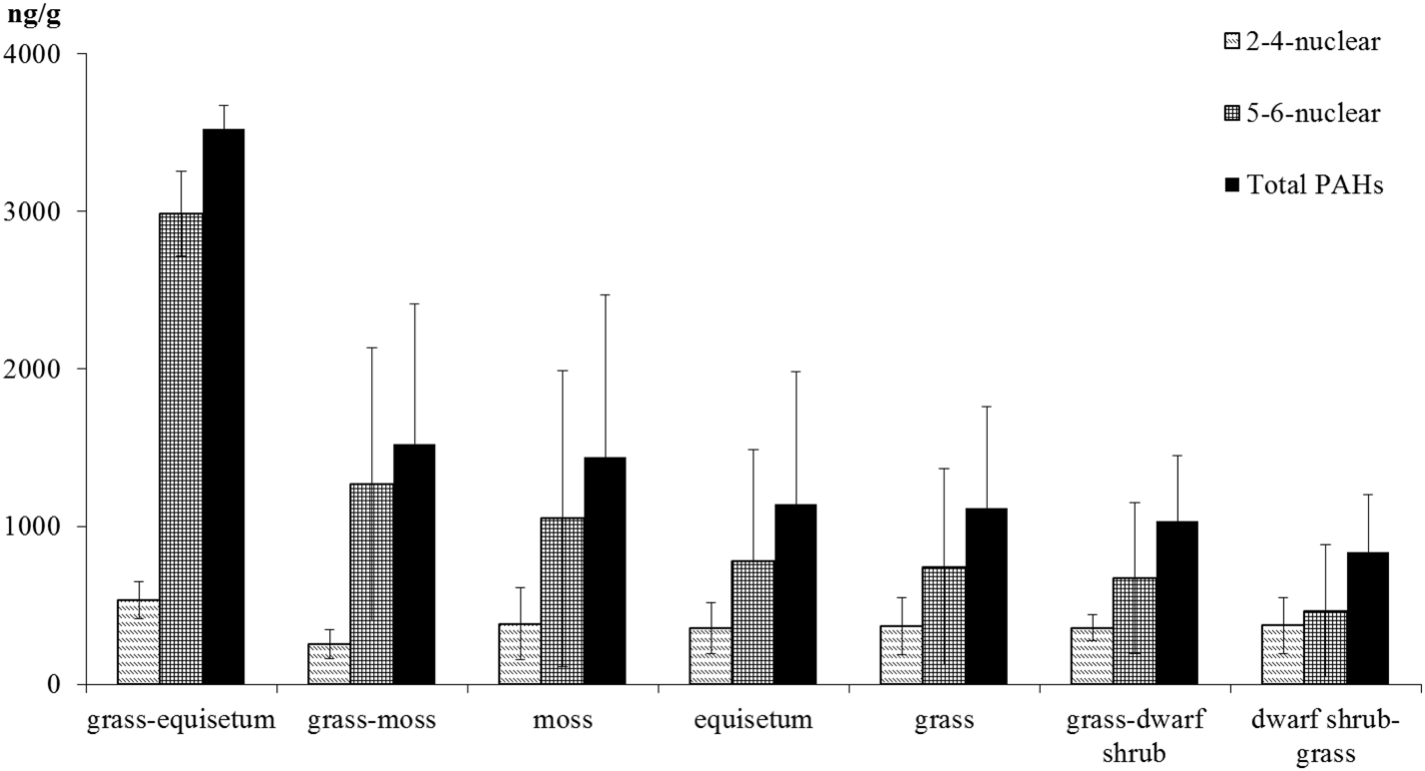
Distribution of low-molecular-weight (light) and high-molecular-weight (heavy) PAHs in peats of various plant macrofossils.

The quantitative and qualitative composition of PAHs depends on the plant macrofossils and trophicity of the peatlands (Fig. 6).

**Figure 7:**
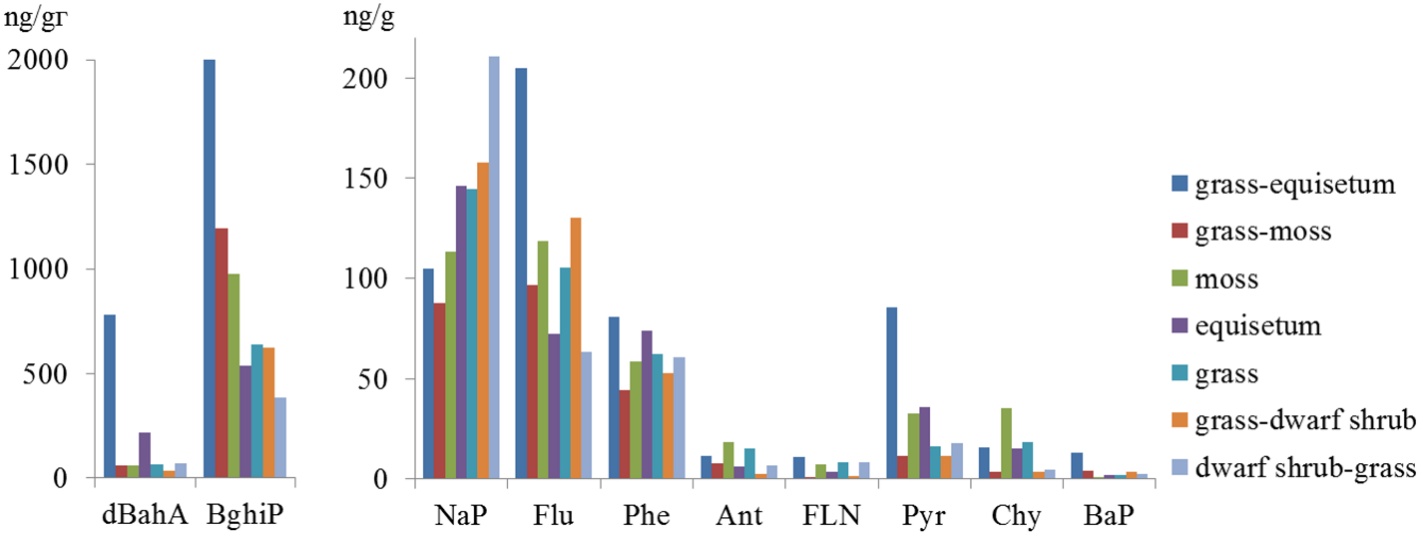
PAH composition in peats of various plant macrofossils.

The grass-equisetum peat contains the maximum of PAHs, the concentration of which is many times higher than of others, mainly due to a very high amount of heavy 5–6-nuclear polyarenes (benzo[*ghi*]perylene and dibenz[*a,h*]anthracene), as well as an increased proportion of 4-nuclear pyrene (Fig. 7). The content of the same PAHs in the equisetum peat is by 2–4 times lower, and in the grass peat—by 3–5 times, respectively.

The subshrub-grass peat contains the minimum of heavy PAHs and the maximum of 2-nuclear naphthalene. In grass-dwarf shrub, grass-moss and moss peat, the contribution of 2-nuclear PAHs (naphthalene and fluorine) as well as 6-nuclear benzo[*ghi*]perylene is the greatest.

### PAH source identification

Among the peat-forming vegetation in the southern shrub tundra, *Betula nana* and *Salix glauca* are the most common species, characterized by low coefficients of biological accumulation of various pollutants: trace elements^68^, polychlorinated biphenyls (PCBs)^69^, as well as aerotechnogenic PAHs coming from coal mining^70^. Polyarenes accumulate at shrubs through the root system and concentrate on the bark surface, whereas the PAH penetration into the stems is minimal^71^. At the peat plateaus of the Eastern European forest tundra, the PAH content is low in *Carex limosa*, and the maximum concentrations of light polyarenes, mainly naphthalene, are found in *Polytrichum strictum*, scions of *Picea abies*, and branches of *Betula pubescens*^50^.

However, plant residues are decomposed by fungi and bacteria in the process of peat formation, which leads to a qualitative change in the composition of PAHs. As a result of microbiological decomposition of plants, heavy PAH structures can be formed from more complex organic compounds contained in these plants, including pentacyclic terpenes, aromatic structures and structures with diene or polyene bonds in aliphatic hydrocarbon chains, lipids^72,73^. The stage of peat decomposition determines the PAH content, depending on plant composition of peat. For instance, in the active layer, there are negative correlations between the PAH composition and peat decomposition stage amounted to R = − 0.81, n = 8, P < 0.05 in peat plateaus and R = − 0.84, n = 10, P < 0.05 if fens^50^. It should be noted that such a high correlation was actually obtained only on the basis of one object: a peat plateau and an adjacent fen. We also found a similar pattern, but only in one area I 2—a peat plateau/thermokarst complex located at the southern limit of the European cryolithozone. Undoubtedly, the stage of peat decomposition can affect the contribution of the PAH composition in plants to the polyarene accumulation in peatlands. However, there is no correlation dependences between the PAH composition of soils and plants depending on the stage of peat decomposition for the entire profile as well as separately for the active and the transition layers.

Over the past few decades, various molecular binary ratios have been applied as chemical indicators to classify probable sources of PAH congeners in soils^32,35,74^. According to the calculation of diagnostic criteria for the PAH genesis, PAHs have not an exclusively petrogenic-biogenic origin in all peatlands. The ratios of phenanthrene/anthracene > 10, fluoranthene/pyrene < 1, fluoranthene/(fluoranthene + pyrene) < 0.5, anthracene/(anthracene + phenanthrene) < 0.1, (pyrene + fluoranthene)/(chrysene + phenanthrene) < 0.5 indicate a petrogenic origin of PAHs, the opposite ratios—a pyrolytic genesis, i.e. the additional contribution of pyrogenic PAHs^74^. The obtained ratios determine a large contribution of pyrogenic PAHs, in particular, an increase in the proportion of anthracene and fluoranthene in some horizons in the studied areas indicates numerous ancient fires. At the same time, naphthalene, acenaphthene, fluorene, pyrene, dibenz[*a,h*]anthracene, and benzo[*ghi*]perylene have a predominantly biopedogenic origin and are formed under anaerobic reducing conditions in water-flooded peatlands.

As stated above, peat plateaus of Western Siberia have a lower content of PAHs, especially heavy polyarenes in the polygonal tundra. This fact is obviously determined by lower mean annual air and ground temperatures, which suppress the vital activity of soil microorganisms producing PAHs, and plants themselves. The maximum accumulation of 6-nuclear benzo[*ghi*]perylene in peat layers is usually an indicator of active decomposition of lignin in herbaceous plants, and among the peat-forming vegetation in polygonal peatlands, dwarf shrubs and mosses predominate. It can be a result of these two reasons, why only 5-nuclear PAHs, in particular dibenz[*a,h*]anthracene, are found in the polygonal peatlands.

### Relationship between the PAH content

**Figure 8:**
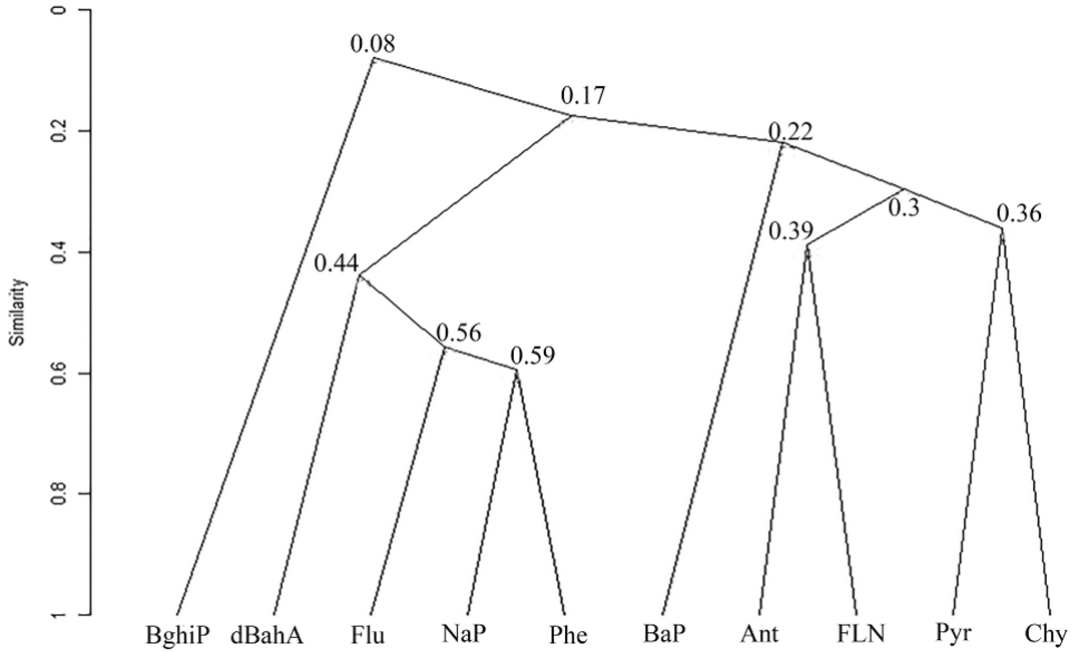
Hierarchical dendrogram of the objects similarity by the Sørensen coefficient.

The conducted cluster analysis showing clustering of studied PAHs according to UPGMA (unweighted pair group method with arithmetic mean) is viewed in Fig. 8. The results of UPGMA have allowed for detecting four separate clusters based on the PAH composition.

The first PAH cluster consists of the most interconnected naphthalene and phenanthrene, as well as fluorene and, to a lesser extent, bound dibenz[*a,h*]anthracene. The second cluster contains a pair of loosely bound anthracene and fluoranthene, as well as a pair of pyrene and chrysene. The second cluster is also most closely related to the only member of the third cluster, benzo[*a*]pyrene. If the first three clusters are weakly interconnected, then the fourth cluster is practically unrelated to them and consists of one member—6-nuclear benzo[*ghi*]perylene. Thus, a small proportion of the similarity of the latter with other PAHs indicates that BghiP does not depend on the concentrations of other PAHs. Concentrations of benzo[*ghi*]perylene are very high and can exceed the sum of all other PAHs, so it is the easiest to quantify when the sensitivity of analytical instruments is low. In addition, as the most highly condensed PAH, BghiP is the most immobilized and stable under an anaerobic environment, although it decomposes rather quickly under aerobic conditions. These facts allow us to conclude that benzo[*ghi*]perylene can be a fairly reliable bioindicator of the conservation of organic matter during the Holocene and in the future.

## Conclusion

Using the high-performance liquid chromatography methods in peat plateaus and polygonal peatlands of the East European and West Siberian cryolithozone, 15 PAHs were identified: naphthalene, acenaphthene, fluorene, phenanthrene, anthracene, fluoranthene, pyrene, benzo[*a*]anthracene, chrysene, benz[*b*]fluoranthene, benz[*k*]fluoranthene, benz[*a*]pyrene, dibenz[*a,h*]anthracene, benzo[*ghi*]perylene, indeno[*1,2,3-cd*]pyrene, the content of which was estimated in a very wide range—112–3673 ng/g with mean 1214 ± 794 ng/g. While the profile distribution pattern of light 2–4-nuclear PAHs is more homogeneous at most sites, the content of heavy 5–6-nuclear PAHs differs widely. At the same time, in East European peat plateaus, the main contribution to the total PAHs is made by 6-nuclear benzo[*ghi*]perylene, the content of which in some layers exceeds the upper determination limit—2000 ng/g; therefore, the concentrations of heavy PAHs are by 4.4 ± 2.8 times higher than those of light polyarenes. In the West Siberian peatlands, benzo[*ghi*]perylene was determined only in interpolygonal cracks and fens, and the maximum contribution to the content of highly condensed PAHs was made by 5-nuclear dibenz[*a,h*]anthracene with mean 86 ± 89 ng/g: on the opposite, the concentration of 2–4-nuclear light PAHs was by 4.0 ± 3.4 times higher than that of heavy 5–6-nuclear polyarenes.

Our results show that the sum of 5–6-nuclear polyarenes depends on the concentration of benzo[*ghi*]perylene by 98%, and the total amount of PAHs—by 95%, whereas the sum of light PAHs is mainly determined by the fractions of phenanthrene and fluorene (R = 0.8 and 0.66, respectively), and is significantly correlated with the C and N content (r = 0.21 and 0.49, respectively).

The qualitative and quantitative composition of PAHs depends on the botanical composition of peat. The maximum content of heavy PAHs was found in grass-equisetum peat, the minimum—in dwarf shrub-grass. In dwarf shrub-grass, grass-moss and moss peat, 2-nuclear PAHs: naphthalene and fluorene as well as 6-nuclear benzo[*ghi*]perylene have the most significant contribution. However, there is no statistically significant correlation between the composition of PAHs in soils and plants, depending on the degree of decomposition of peat, found both for the entire thickness of peat plateau and in its individual horizons.

The lower amounts of PAHs in the West Siberian peatlands compared to the East European ones are explained by two reasons. The relatively low air and soil temperatures reduce the vital activity of soil microorganisms that produce PAHs, and also affect the species composition of the peat-forming plants themselves. In particular, 6-core benzo[*ghi*]perylene in peat layers usually accumulates as a result of lignin decomposition of herbaceous plants, but among the peat-forming plants in polygonal peatlands, dwarf shrubs and mosses predominate.

The cluster analysis of PAH similarity showed that the content of 6-nucleus benzo[*ghi*]perylene is independent of the concentrations of other polyarenes. Thus, benzo[*ghi*]perylene being the most decomposition-resistant polyarene in an anaerobic environment, could be a marker of accumulation and retention of organic matter during periods of the Holocene and predicted permafrost thaw, since anaerobic conditions persist with excessive moisture.

## Data Availability

The datasets used during the current study are available from the corresponding author on reasonable request.
